# Stem-like breast cancer cells in the activated state resist genetic stress via TGFBI-ZEB1

**DOI:** 10.1038/s41523-021-00375-w

**Published:** 2022-01-13

**Authors:** Qi Sun, Yufen Wang, Adam Officer, Brianna Pecknold, Garrett Lee, Olivier Harismendy, Jay S. Desgrosellier

**Affiliations:** 1 Department of Pathology, University of California, San Diego, La Jolla, CA 92093 USA; 2 Moores Cancer Center, University of California, San Diego, La Jolla, CA 92093 USA; 3 Department of Medicine, University of California, San Diego, La Jolla, CA 92093 USA; 4 Present Address: Department of General Surgery, The First Affiliated Hospital of Xi’an Jiaotong University, Xi’an, 710061 China

**Keywords:** Tumour heterogeneity, Cancer stem cells, Breast cancer, Cancer stem cells, Targeted therapies

## Abstract

Breast cancer cells with stem-like properties are critical for tumor progression, yet much about these cells remains unknown. Here, we characterize a population of stem-like breast cancer cells expressing the integrin αvβ3 as transcriptionally related to activated stem/basal cells in the normal human mammary gland. An unbiased functional screen of genes unique to these cells identified the matrix protein TGFBI (BIG-H3) and the transcription factor ZEB1 as necessary for tumorsphere formation. Surprisingly, these genes were not required for cell proliferation or survival, but instead maintained chromosomal stability. Consistent with this finding, CRISPR deletion of either gene synergized with PARP inhibition to deplete αvβ3^+^ stem-like cells, which are normally resistant to this therapy. Our findings highlight a critical role for TGFBI-ZEB1 protection against genetic stress as a key attribute of activated stem-like cells and suggest that disrupting this ability may enhance their “BRCAness” by increasing sensitivity to PARP inhibitors.

## Introduction

Tumor-initiating cancer stem cells (CSCs) bearing similarities to adult mammary stem cells (MaSCs) are important contributors to breast cancer progression and metastasis^1–4^. However, adult MaSCs are highly dynamic, frequently changing their cell state—a physiological condition due to altered gene expression or signaling—in response to hormonal cues. In fact, the mammary gland is one of the most dynamic organs in adult women, undergoing robust epithelial remodeling in response to hormones during the menstrual cycle and pregnancy that is driven by stem cells. While normally quiescent, MaSCs respond to hormones indirectly via paracrine signals to become active and contribute to epithelial remodeling^5–8^ since they lack hormone receptors^9^. These active stem cells exhibit enhanced proliferation and migration^5–8^, features that make this signaling state likely to be hijacked by tumor cells. This raises the tantalizing question of whether some of the most aggressive CSCs may further acquire properties associated with activated stem cells.

We previously showed that the cell surface receptor integrin αvβ3 is a key switch turned-on by activated stem cells as they are mobilized for epithelial remodeling during pregnancy^10^. Using αvβ3 as a marker, we further characterized a unique and particularly aggressive population of stem-like breast cancer cells^11^. Unexpectedly, we found αvβ3^+^ CSCs in aggressive patient tumors that were either estrogen receptor-positive (ER^+^), human epidermal growth factor receptor-positive (HER2^+^), or triple-negative^11^, suggesting these cells may contribute to disease progression in all clinical subtypes. Notably, αvβ3 expression was not synonymous with traditional CSC profiles, such as CD44^+^/CD24^Low^^1^, CD49f^+^/EpCAM^Low^^2^, the claudin-low intrinsic subtype^12^ or mesenchymal markers^2,13,14^. Instead, αvβ3^+^ cells represented a distinct subset of these broader classifications^11^. Our prior findings provided valuable insight regarding the aggressive nature of αvβ3^+^ CSCs and emphasized the need to further elucidate the unique genes and signaling pathways required for their function.

Despite the importance of CSCs for breast cancer progression, studies of these cells are limited by their scarcity and a lack of appropriate cell line models that reflect the heterogeneity in a patient tumor. In the present study, we make use of our previously characterized heterogeneous breast cancer cell line models to overcome this limitation. These cell lines better recapitulate the intratumoral heterogeneity in patient disease^2,15^, including a subset of αvβ3^+^ CSCs, and allow us to directly assess a role for these cells compared to other neighboring tumor cell types. Based on our prior findings, we hypothesized that tumor cells bearing αvβ3 may similarly express genes found in stem/basal cells in response to hormonal signaling during the menstrual cycle or pregnancy. Furthermore, since αvβ3 is a biomarker of aggressive cancer cells, we propose that these cells may contain unique genes/pathways that could serve as potential vulnerabilities. To address these questions, we performed unbiased whole transcriptome analysis of αvβ3^+^ CSCs. These findings represent an initial step toward revealing similarities between these cells and normal mammary cell types and identifying key pathways that may control their aggressive behavior.

## Results

### Surface αvβ3 marks stem-like cells enriched for tumor initiation

Breast cancers are heterogeneous, with cells representing different mammary lineages often found in the same tumor, including those with stem-like properties^14,16^. We previously showed in patient breast cancers that cells expressing the surface marker integrin αvβ3 represent a stem-like cancer cell subset associated with disease progression in a diverse array of subtypes^11^. Despite the potential significance of αvβ3^+^ CSCs for disease progression, few good models exist to study these cells in the context of other non-stem cells. Additionally, the scarcity of these cells in patient samples represents another practical limitation to studying these cells. One potential in vitro model for our studies is the heterogeneous HCC38 cell line, which consists of luminal-like (CD49f^+^/EpCAM^high^) and stem-like cell types (CD49f^+^ EpCAM^low^)^13^. Our analysis of surface αvβ3 in these cells further identified a population of EpCAM^Low^/αvβ3^+^ cells enriched for stemness properties such as tumorsphere formation and self-renewal^11^. Thus, to more closely reflect the situation in patients’ tumors, we examined the HCC38 breast cancer cell line as a potential model for our studies of αvβ3^+^ CSCs.

**Fig. 1:**
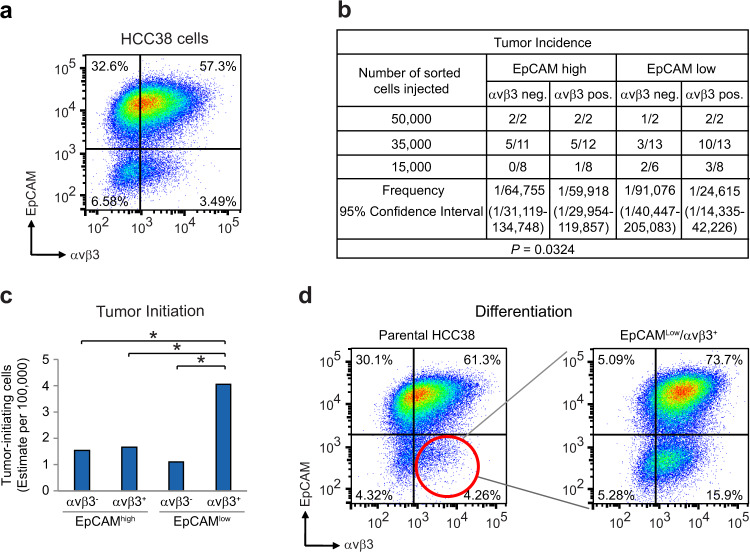
Integrin αvβ3 enriches for tumor-initiating ability and stem-like properties. **a** Representative FACS density plot of HCC38 breast cancer cells showing the live, CD49f^+^ cells according to their cell surface EpCAM and αvβ3 status. **b** Table describing the frequency of tumor formation per fat pad injected for each sorted cell type. Results pooled from four independent experiments. **c** Histogram showing the estimated number of tumor-initiating cells from the data in (**b**). **b**, **c** Statistics by Extreme Limiting Dilution Analysis (ELDA), which uses a chisquare likelihood ratio test to calculate *p*-values between groups. *P < 0.05. **d** Representative FACS density plots showing differentiation of sorted EpCAM^Low^/αvβ3^+^ cells re^-^analyzed after 6 weeks (10 passages). **a**, **d** *n* = 3 independent experiments. See also Supplementary Fig. 1.

To rigorously compare stemness traits in vivo, we sorted HCC38 cells into four populations based on their EpCAM and αvβ3 status (Fig. 1a and Supplementary Fig. 1a) prior to evaluating their tumor-initiating potential in vivo (Fig. 1b, c). Sorted cells were injected orthotopically into the inguinal mammary gland fat pads of adult female immunocompromised mice, then compared for their ability to initiate new tumors in limiting dilution assays (Fig. 1b, c). We now show that EpCAM^Low^/αvβ3^+^ cells possess about a 4-fold greater ability to initiate tumors relative to other HCC38 cell types (Fig. 1b, c). This is consistent with our prior tumorsphere results^11^ and further supports their characterization as stem-like cells. Another important attribute of stem-like cells is their ability to differentiate. To determine if EpCAM^Low^/αvβ3^+^ cells also possessed this property we cultured sorted cells for exactly 10 passages prior to re-analyzing by flow cytometry (Fig. 1d and Supplementary Fig. 1b). This showed that indeed these cells were capable of differentiating into all three of the other cell types analyzed. Comparison with parental HCC38 cells showed that there was a great deal of lineage specificity with regards to each sorted cell type, with EpCAM^Low^/αvβ3^+^ cells displaying a preference for differentiating into EpCAM^High^/αvβ3^+^ cells (Fig. 1d and Supplementary Fig. 1b). Interestingly, the EpCAM^High^/αvβ3^+^ cells changed the least, suggesting that they represent a more stable differentiated cell type (Supplementary Fig. 1b). Thus, similar to patients’ tumors, we show that the HCC38 cell line contains a rare subset of αvβ3^+^ CSCs, in addition to non–stem cell types, representing an ideal in vitro model to begin parsing critical gene expression and signaling differences.

### αvβ3^+^ CSCs express genes associated with activated stem cells

**Fig. 2:**
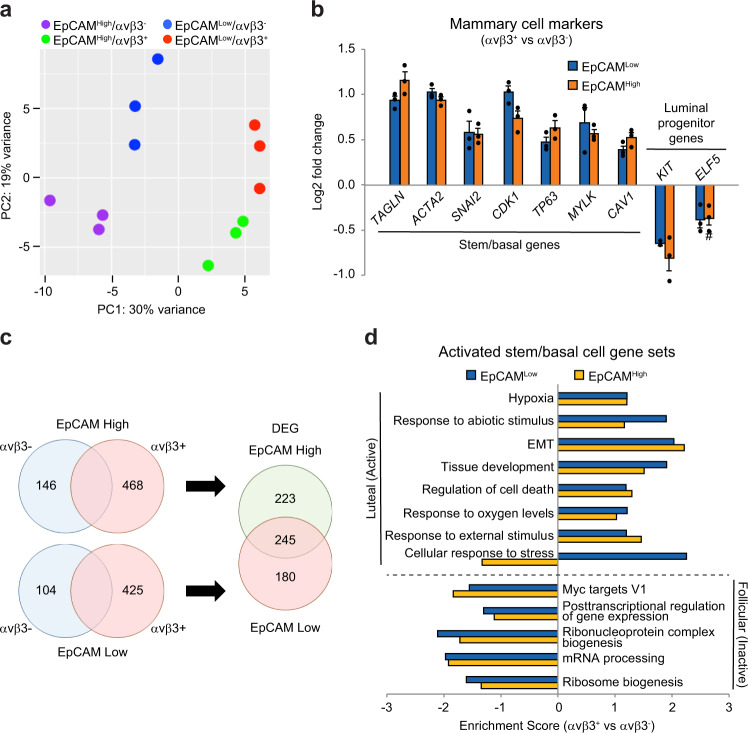
αvβ3^+^ CSCs are similar to activated stem/basal cells from the normal human mammary gland. **a** Principal component analysis (PC) performed on bulk RNA-Seq data from each of the indicated HCC38 sorted cell types. **b** Relative expression of select gene markers of stem/basal or luminal progenitor cells in αvβ3^+^ versus αvβ3^−^ cells. Data represent the mean ± s.e.m. # = not significant. **c** Venn diagrams depicting the number of differentially expressed genes (DEG) identified in each cell type. The selection criteria was ≥1.5-fold change in gene expression and *P* < 0.05. **d** Comparison of αvβ3^+^ versus αvβ3^−^ cell GSEA results with the top gene sets enriched in stem/basal cells during luteal (Active) versus follicular (Inactive) menstrual cycle phases. **b**, **d** Statistics by Student’s t-test with Benjamini-Hochberg multiple comparisons test. **a**–**d** *n* = 3 independent experiments. See also Supplementary Fig. 2.

To discover critical stemness genes in αvβ3^+^ CSCs, and determine any similarities with normal mammary cell types, we performed bulk RNA-Seq analysis. The principal component analysis highlighted a surprising amount of distinction between αvβ3^+^ and αvβ3^-^ cells (Fig. 2a), even greater than that due to EpCAM status alone, a widely-used marker to identify stem-like cells^2^. This was even more surprising since EpCAM^high^ and EpCAM^Low^ cell types are widely separated and distinct populations, whereas αvβ3 expression represented a continuum of high and low expressers (Fig. 1a). Meanwhile, both αvβ3^+^ cell types exhibited a high degree of similarity at the transcriptional level (Fig. 2a). This suggests a potential relationship between these cell types, consistent with our differentiation results (Fig. 1d). To probe this relationship further we compared the expression of a few select markers of normal mammary cell types. Since αvβ3 expression has previously been shown to occur on both stem/basal and luminal progenitor cells in the normal murine and human mammary gland^10,17,18^ we examined markers previously established to differentiate between these two cell types^16^. Our analysis of these mammary cell markers showed that both αvβ3^+^ cell types are enriched for genes associated with stem/basal, but not luminal progenitor cells (Fig. 2b and Supplementary Fig. 2a), consistent with our hypothesis that αvβ3^+^ CSCs display characteristics of adult MaSCs.

To further probe any potential similarity between αvβ3^+^ CSCs and activated stem/basal cells from the normal mammary gland we assessed the differentially expressed genes (DEG) within each cell type (Fig. 2c) and performed gene set enrichment analysis (GSEA). While αvβ3^+^ cell types were closely related (Fig. 2a, b), we identified 180 genes enriched in EpCAM^Low^/αvβ3^+^ cells compared to EpCAM^High^/αvβ3^+^ cells (Fig. 2c and Supplementary Fig. 2b). We then compared gene sets enriched in both αvβ3^+^ cell types with those from activated stem/basal cells in the normal human mammary gland. Since data from pregnancy is unavailable, we compared our GSEA results with published data from normal basal/stem cells during the luteal (Active) versus follicular (Inactive) phases of the menstrual cycle^19^. Many of the same hormone-induced changes that occur during the luteal phase also happen during pregnancy. The results were striking, as gene sets found in activated stem/basal cells were overwhelmingly shared by αvβ3^+^ cancer cells, while those in inactive cells were not (Fig. 2d and Supplementary Fig. 2c). Interestingly, of the gene sets analyzed, the αvβ3^+^ cells differed only in the genes involved in the cellular response to stress, with this representing a unique feature distinguishing EpCAM^Low^/αvβ3^+^ cells (Fig. 2d). These findings highlight an association between αvβ3^+^ CSCs and the activated state in normal mammary stem/basal cells and suggest that a heightened response to stress may be a key distinguishing feature of these cells.

### Identification of key genes unique to αvβ3^+^ CSCs

**Fig. 3:**
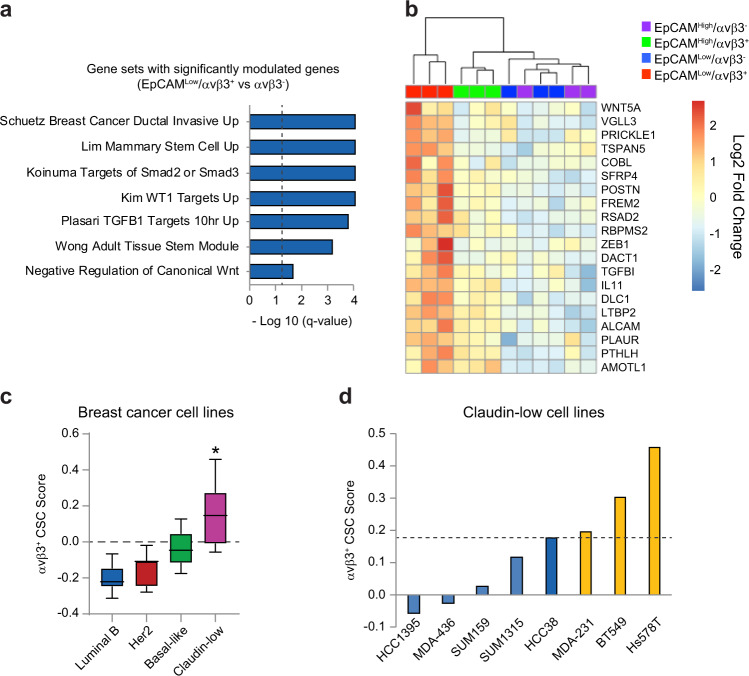
αvβ3^+^ CSCs express unique genes associated with aggressive breast cancers and normal mammary stem cells. **a** Gene set enrichment analysis (GSEA) for EpCAM^Low^/αvβ3^+^ versus αvβ3^−^ cells. Statistics by Student’s t-test with Benjamini−Hochberg test. Dashed line indicates statistical significance. **b** Heat map of the 20 most differentially expressed genes upregulated in EpCAM^Low^/αvβ3^+^ cells and found in the gene sets in (**a**) (Log2 scale). **c** Box and whiskers plot showing the relative enrichment for the αvβ3^+^ CSC gene signature in cell lines representing different molecular subtypes. Boxplots represent medians (center line) and interquartile range (IQR; box), and whiskers represent the maximum and minimum values within 1.5 times the IQR from the edge of the box. Statistics by ANOVA with Tukey’s test. *P < 0.05. Cell lines in each category: Luminal B; *n* = 7, HER2; *n* = 7, Basal-like; *n* = 6, Claudin-low; *n* = 8. **d** Claudin-low cells lines with significant enrichment for the αvβ3^+^ CSC gene signature compared to parental HCC38 cells (dashed line). **a**–**d** *n* = 3 independent experiments. See also Supplementary Fig. 3.

Based on our findings that αvβ3^+^ CSCs enrich for stemness properties such as tumor initiation (Fig. 1b, c) we wished to determine the key genes and signaling pathways critical for their function. We began by selecting several gene sets associated with αvβ3^+^ CSCs based on their relevance to breast cancer, stem cells, or signaling pathways (Fig. 3a and Supplementary Fig. 3a). By determining which of the 180 DEG’s identified in Fig. 2c were present within each gene set, we identified 20 candidate genes unique to αvβ3^+^ CSCs (Fig. 3b and Supplementary Fig. 3b), referred to as our αvβ3^+^ CSC signature. In order to perform a functional screen of these genes, we sought to identify appropriate surrogate cell lines for our αvβ3^+^ CSCs. For this analysis, we made use of published gene sets from 28 breast cancer cell lines that were previously used to classify these cells according to their intrinsic subtype^13^. Comparison with our αvβ3^+^ CSC signature revealed specific enrichment in the claudin-low cell type (Fig. 3c), consistent with prior characterization of these cells as stem-like^11^. However, careful analysis of each of the eight claudin-low cell lines revealed that only three of them displayed any enrichment beyond the parental HCC38 cells (Fig. 3d), in which αvβ3^+^ CSCs are only a small fraction of the total cells. The three cell lines identified (MDA-MB-231, BT549, and Hs578T) represent some of the most widely used breast cancer cell lines due to their high tumorigenicity and metastatic potential, highlighting the potential significance of our candidate genes for aggressive disease. These findings serve to distinguish αvβ3^+^ CSCs as a unique cancer cell type that is not synonymous with the claudin-low classification and identify appropriate surrogate cell lines in which to screen our candidate genes for their role in stemness.

### Characterization of ZEB1 and TGFBI as candidate genes required for stemness

**Fig. 4:**
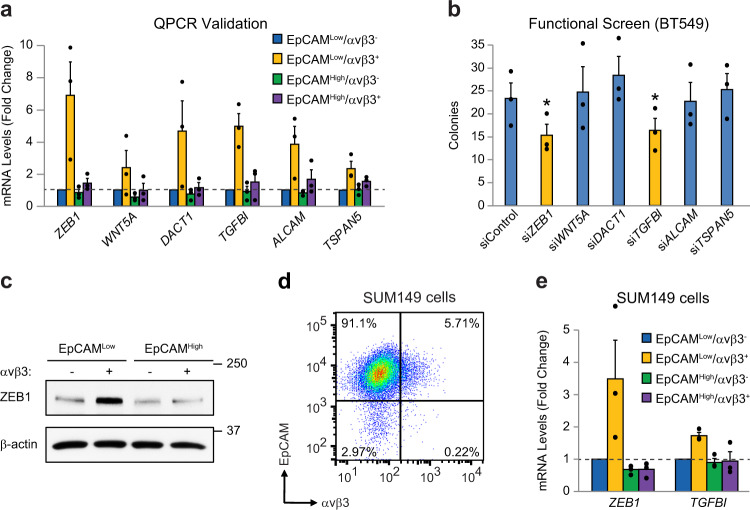
Identification of TGFBI and ZEB1 as candidate genes unique to αvβ3^+^ CSCs. **a** QPCR validation of candidate genes in sorted HCC38 cell types. **b** Functional screen for candidate genes necessary for methylcellulose colony formation after transient siRNA knockdown in BT549 cells. Target gene knockdown was validated by QPCR. Statistics by one-way ANOVA with Dunnett’s test. *P < 0.05. **c** Representative immunoblot of lysates from sorted HCC38 cells. β-actin is shown as a loading control. Molecular weight markers are indicated in kilodaltons. **d** Representative FACS density plot of the live, CD49f^+^ SUM149 cells according to their cell surface EpCAM and αvβ3 status. **e** QPCR validation of candidate genes in sorted SUM149 cells. **a**, **e** Samples were run in duplicate with GAPDH as a loading control. Expression is shown relative to the EpCAM^Low^/αvβ3^−^ cells (dashed lines). **a**, **b**, **e** Data represent the mean ± s.e.m. **a**–**e** *n* = 3 independent experiments. See also Supplementary Fig. 4.

We next wished to perform an unbiased assessment of the key genes and signaling pathways responsible for the more aggressive nature of αvβ3^+^ CSCs. Here, we used αvβ3 as a marker of activated stem-like cells, with candidate genes selected without regard for a potential direct link to αvβ3 signaling. To identify candidate genes, we performed QPCR analysis of the 20 DEGs from Fig. 3b to select those that displayed the most consistent and robust expression in αvβ3^+^ CSCs relative to the other three cell types (Fig. 4a). This identified six genes for further analysis (Fig. 4a). We then performed an siRNA functional screen to identify which of these genes was most critical for αvβ3^+^ CSCs (Fig. 4b and Supplementary Fig. 4a). To simultaneously examine the function of multiple genes after transient siRNA knockdown we employed the BT549 cell line as an appropriate surrogate for our αvβ3^+^ CSCs, as characterized in Fig. 3d. Tumorsphere formation in methylcellulose was selected as our primary endpoint since it is a critical stemness property. Results from these studies identified two genes as highly relevant for further study: *TGFBI* (Transforming Growth Factor Beta Induced; initially termed BIG-H3) and *ZEB1* (Fig. 4b and Supplementary Fig. 4a). We rigorously validated these targets by showing ZEB1 protein enrichment in sorted αvβ3^+^ CSCs from HCC38 cells (Fig. 4c). Additionally, we used another heterogeneous cell line (SUM149) to show conserved expression of both *TGFBI* and *ZEB1* specifically in EpCAM^Low^/αvβ3^+^ cells (Fig. 4d, e and Supplementary Fig. 4b). Importantly, ZEB1 protein levels were specifically associated with the surrogate cell lines enriched for the αvβ3 CSC gene signature (Supplementary Fig. 4c), as well as the LM2-4 metastatic variant of the MDA-MB-231 cell line^20^ (Supplementary Fig. 4d), all of which we previously showed to express αvβ3^11^.

A secreted ECM protein, TGFBI (Transforming Growth Factor Beta Induced; BIG-H3) has been shown to paradoxically enhance anchorage-independence^21^, similar to our findings with αvβ3^22^. While best known for its role in epithelial-mesenchymal transformation (EMT)^23^, the transcription factor ZEB1 also mediates non-EMT functions that may be more important for its role in tumor progression^24,25^. In fact, recent studies unexpectedly found ZEB1 in a subset of basal/stem cells in normal human mammary glands^26^ where it promotes oncogene-induced transformation^24^. While the exact identity and function of these cells is still a mystery, it suggests they may be similar to ZEB1^+^ breast cancer cells.

### Discovery of TGFBI-ZEB1 as a key stemness-related signaling module

**Fig. 5:**
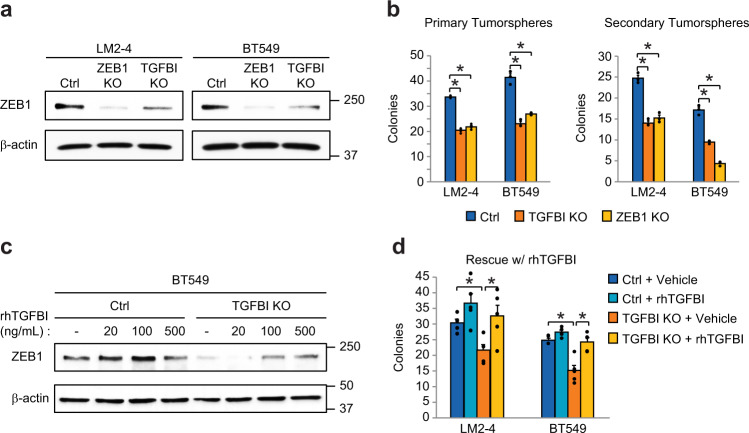
Discovery of a TGFBI-ZEB1 signaling module required for stemness. **a** Representative immunoblot of lysates from TGFBI and ZEB1 CRISPR knockout cells. **b** Tumorsphere assays in methylcellulose to assess the effects of TGFBI or ZEB1 CRISPR knockout on primary colonies (left) or self-renewal (right) in the indicated cell lines. **c** A representative immunoblot experiment, showing rescue of ZEB1 protein levels after treatment with rhTGFBI for 24 h with the indicated doses compared to vehicle control (PBS). **a**, **c** β-actin is shown as a loading control. Molecular weight markers are indicated in kilodaltons. **d** Rescue of primary colony formation in TGFBI knockout cells with 500 ng/mL rhTGFBI. Data represent the mean ± s.e.m. Statistics by one-way (**b**) or two-way (**d**) ANOVA with Dunnett’s (**b**) or Tukey’s test (**d**). *P < 0.05. *n* = 3 (**a**–**c**) or *n* = 5 (**d**) independent experiments. See also Supplementary Fig. 5.

In order to further assess the relevance of these two genes for stemness properties, we generated TGFBI and ZEB1 knockout cells using CRISPR/Cas9 in our surrogate stem-like LM2-4 and BT549 cell lines. We further validated these cells, showing significantly reduced ZEB1 protein levels (Fig. 5a) as well as decreased amounts of TGFBI mRNA expression (Supplementary Fig. 5a) and secreted TGFBI protein (Supplementary Fig. 5b). Surprisingly, these validation studies showed that TGFBI deletion also resulted in decreased levels of ZEB1 protein (Fig. 5a). Importantly, deletion of ZEB1 did not decrease levels of TGFBI mRNA (Supplementary Fig. 5c). We also noted that deleting either gene had no effect on protein levels of the β3 subunit (Supplementary Fig. 5d). These unexpected findings suggest that these two independently identified candidate genes are linked within the same pathway

To examine this possibility and validate their role in stemness, we tested our TGFBI and ZEB1 CRISPR knockout cells in assays of primary tumorsphere formation and self-renewal (Fig. 5b). Deletion of either TGFBI or ZEB1 resulted in an approximately 50% decrease in primary tumorspheres, while subsequent self-renewal assays showed an almost 75% decrease due to ZEB1 knockout in BT549 cells (Fig. 5b). These findings highlight an important role for these genes in stemness and are consistent with their function within the same pathway. Indeed, we show that adding recombinant human TGFBI protein (rhTGFBI) is sufficient to drive ZEB1 protein expression in control and TGFBI knockout cells (Fig. 5c) and specifically rescue defective tumorsphere formation caused by TGFBI deletion (Fig. 5d). Our findings highlight a potential new TGFBI-ZEB1 signaling module specific to αvβ3^+^ CSCs identified by a rigorous, unbiased and systematic approach.

### TGFBI-ZEB1 promotes chromosomal stability and resistance to PARP inhibition

We next considered the cell biological basis for these effects on tumorsphere formation. Our GSEA results suggest that the ability to respond to cellular stress was a distinguishing feature of αvβ3^+^ CSCs. In fact, while TGFBI and ZEB1 have diverse cellular functions, they may play a common role in reducing a certain type of genetic stress called chromosomal instability (CIN)^24,27^. CIN is a hallmark of cancer and an important stress in cancer cells that limits transformation^24^. In fact, a recent study showed that normal adult MaSCs were inherently more tumorigenic due to suppression of CIN via ZEB1^24^. Our independent discovery of ZEB1 as one of the most DEG in αvβ3^+^ CSCs, suggested that it may play a similar role in these cells.

**Fig. 6:**
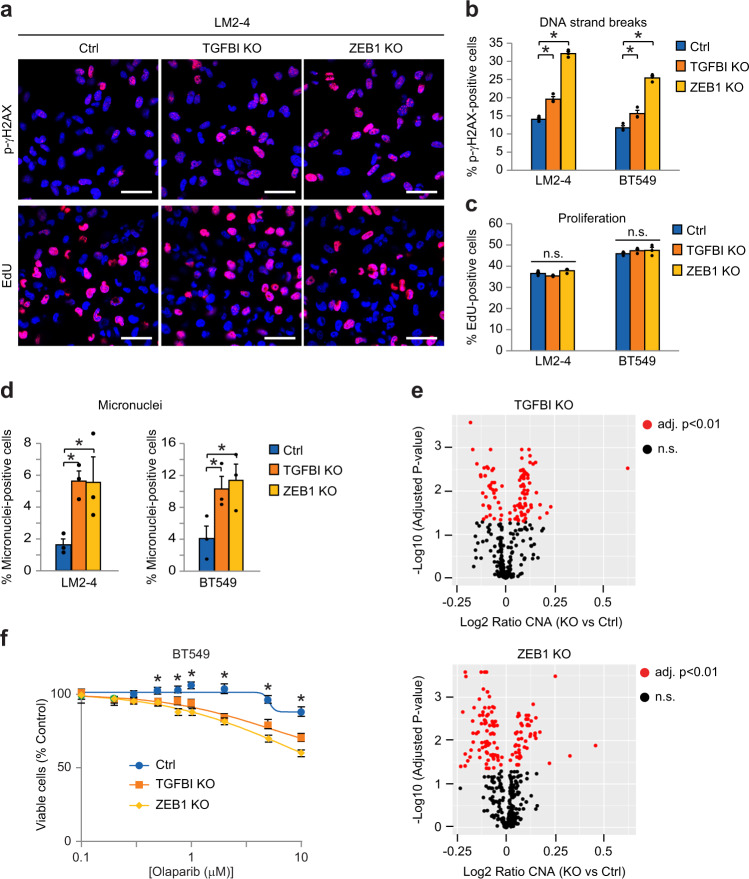
Decreased TGFBI-ZEB1 signaling enhances chromosomal instability and sensitivity to PARP inhibition. **a** Representative immunofluorescent staining for the DNA damage marker phospho-γH2AX (red) or detection of fluorescently-labeled EdU (red) after 90 min incubation to assess cell proliferation. Nuclei are stained blue in all images. Scale bars, 40 μm. Percentage of cells positive for p-γH2AX (**b**), EdU (**c**), or micronuclei (**d**) relative to total nuclei. Data calculated from four random fields per condition for each experiment. **e** Volcano plots depicting the copy number alterations (CNA) in BT549 TGFBI or ZEB1 knockout cells relative to controls. Statistics performed by Student’s t-test corrected for multiple testing using the Benjamini-Hochberg method. Red dots represent the 112 (TGFBI KO) and 129 (ZEB1 KO) segments with statistically significant differences (adjusted *p*-value < 0.01) out of 459 total segments examined. Black dots are not significant (n.s.). **f** XTT cell viability assays comparing Olaparib sensitivity in the indicated BT549 cell types. Curves are plotted relative to vehicle controls for each group and fitted by non-linear regression. *P < 0.05 for 0.5, 0.75, 1, 2, 5, and 10 μM Olaparib (TGFBI or ZEB1 KO versus control). *n* = 3 (**a**–**d**, **f**) or *n* = 4 (**e**) independent experiments. Data represent the mean ± s.e.m. Statistics by one-way (**b**−**d**) or two-way (**f**) ANOVA with Dunnett’s test. *P < 0.05. n.s.= not significant. See also Supplementary Fig. 6.

Using multiple methods, we now show that CRISPR knockout of ZEB1 or TGFBI enhances CIN in stem-like cell lines. We began by measuring staining for the DNA damage marker phospho-γH2AX and found that DNA strand breaks increased in our knockout cells (Fig. 6a, b). In contrast, there was no effect on proliferation as assessed by incorporation of fluorescently-labeled EdU (Fig. 6a, c) or apoptosis measured by PARP cleavage (Supplementary Fig. 6a). Since staining with phospho-γH2AX indicates the presence of DNA strand breaks that could lead to missegregation of chromosomes, we quantified micronuclei as a direct measure of CIN and observed increased levels associated with both knockout cell lines (Fig. 6d). To robustly examine differences in CIN we also evaluated potential copy number alterations (CNA) and found higher levels in our knockout cells (Fig. 6e). Analysis of data from The Cancer Genome Atlas further supported these findings by showing that high ZEB1 expression in tumors corresponded with low levels of CIN (Supplementary Fig. 6b). Thus, TGFBI-ZEB1 signaling appears to promote stemness in αvβ3^+^ CSCs by suppressing the endogenous genetic stress caused by CIN.

Tumor cells with defective DNA repair due to BRCA mutations are highly sensitive to PARP inhibition due to an accumulation of double-strand breaks that tips the balance toward cell death^28^. Since we observed increased DNA strand breaks in our TGFBI and ZEB1 CRISPR knockout cells (Fig. 6a, b), we hypothesized that some of these may fail to be repaired, possibly leading to synergy with PARP inhibitors such as Olaparib (Lynparza). While Olaparib is clinically-approved for BRCA-mutant breast and ovarian cancers, non-BRCA mutant cancers are completely refractory to this treatment^29^. Indeed, we show that while deletion of either TGFBI or ZEB1 had no effect on 2D cell viability (Supplementary Fig. 6c), knockout of either gene synergized with Olaparib in two different stem-like cell lines (Fig. 6f and Supplementary Fig. 6d). Significantly, we observed sensitivity to Olaparib at similar doses that are effective against a BRCA-mutant cell line (Supplementary Fig. 6e). These data are consistent with an important role for TGFBI-ZEB1 in reducing CIN in αvβ3^+^ CSCs, highlighting the ability to control genetic stress as a critical attribute of stem-like cells. Additionally, our findings suggest that PARP inhibition may be an effective precision therapy for more than just BRCA-mutant disease, and that a similar approach may be able to eliminate αvβ3^+^ CSCs and reduce breast cancer progression.

## Discussion

While stem cells in the adult mammary gland are dynamic, cycling through active and inactive cell signaling states due to hormonal signaling^10,30,31^, it is unclear if stem-like tumor cells possess a similar ability. Our prior work identified the integrin αvβ3 as a surface marker of activated stem cells in the adult mammary gland, suggesting that tumor cells expressing this marker may feature a similar activated signaling state. By comparing our whole transcriptome sequencing data from sorted αvβ3^+^ CSCs with published gene sets enriched in stem/basal cells during the luteal (Active) and follicular (Inactive) phases of the menstrual cycle, we now show a striking correlation between αvβ3^+^ CSCs and activated stem/basal cells from the normal human mammary gland. We further identified key genes associated with this cell state including the secreted matrix protein *TGFBI* (BIG-H3) and the transcription factor *ZEB1*. Taken together, our findings suggest that these genes may operate as a TGFBI-ZEB1 signaling module to promote stemness by protecting against genetic stress, such as CIN. In fact, downregulating TGFBI-ZEB1 sensitized αvβ3^+^ CSCs to PARP inhibition, laying the foundation for a potential new treatment strategy to reduce breast cancer progression.

Unbiased analysis of critical genes and pathways in αvβ3^+^ CSCs led to our surprising discovery of a TGFBI-ZEB1 signaling module. While TGFBI is a secreted ECM protein that would normally bind to integrins and elicit adhesion-dependent responses, we and others have now shown that it can also enhance anchorage-independent growth^21^. This is similar to our surprising finding that the integrin αvβ3 also promotes anchorage-independence^22^, and suggests the two may function as a possible ligand-receptor pair in stem-like cells. The transcription factor ZEB1 is perhaps best known for its role in EMT; however, it is also important for functions not related to EMT that may be even more critical for tumor progression^24,25^. An unexpected result of gene atlas studies from the normal human mammary gland was the discovery of a subset of stem/basal cells expressing ZEB1^26^. Notably, these cells were specific to human glands and not observed in mice^23^. Further corroborating this finding, a different study identified ZEB1 expression in enriched populations of human MaSCs, where it surprisingly functioned to promote oncogene-induced transformation^24^. Thus, in the normal mammary gland, ZEB1 is expressed in cells that display stem cell properties. While there is still much to learn about these cells, our new findings suggest they may represent MaSCs in the activated state and display traits similar to ZEB1^+^ breast cancer cells. Together, our results highlight a potential new TGFBI-ZEB1 pathway specific to αvβ3^+^ CSCs that we identified through a rigorous, unbiased and systematic approach.

While CIN is a hallmark of cancer cells, too much may act to limit tumor progression^24^. In fact, normal cell types that can better tolerate CIN, such as MaSCs are much more likely to undergo oncogenic transformation^24^, suggesting that control of genetic stress is an important attribute of more aggressive tumor cells. While TGFBI and ZEB1 have diverse cellular functions, they may play a common role in reducing CIN^24,27^. In fact, a recent study showed that MaSCs were inherently more tumorigenic due to suppression of CIN via ZEB1^24^. Our independent discovery of ZEB1 as one of the most differentially-expressed genes in αvβ3^+^ CSCs, suggested that it may play a similar role. We now show that CRISPR knockout of either ZEB1 or TGFBI increased CIN in two stem-like cell lines with no effect on cell proliferation or survival. This genetic instability may be caused by endogenous factors such as cell replication or the production of reactive oxygen species during metabolism. The latter of which would be consistent with the enhanced metabolic activity identified in stem-like breast cancer cells found in metastases^32^. Thus, our investigation of the biological effects of TGFBI-ZEB1 support a role in limiting the effects of genetic stress and maintaining chromosomal stability, suggesting this may be a defining attribute and potential vulnerability of stem-like cells.

PARP inhibitors are clinically-approved and highly effective treatments for BRCA-mutant breast and ovarian cancers^28^. Tumor cells with defects in DNA double-strand break repair, such as BRCA mutations, are more sensitive to PARP inhibitors, such as Olaparib, which prevent single-strand break repair and drive further genetic instability, resulting in cell death. However, while PARP inhibition is an effective treatment against BRCA-mutant breast and ovarian cancers, non-BRCA mutant cancers are completely refractory to this treatment^29^. We now provide proof-of-concept that disrupting TGFBI-ZEB1 signaling not only increased CIN, but functioned much like a BRCA mutation by increasing sensitivity to PARP inhibition. We hypothesized that this may lead to synergy with Olaparib, which normally affects only BRCA-mutant cancers. Indeed, our new data shows that TGFBI or ZEB1 deletion enhances “BRCAness”, and synergizes with Olaparib. Our findings suggest that disrupting key mediators of chromosomal stability in αvβ3^+^ CSCs, such as TGFBI and ZEB1, can sensitize these normally resistant cells to treatment with clinically-approved PARP inhibitors. Therefore, these findings represent a crucial initial step laying the foundation for further study of the activated stem cell state as a key contributor to recurrence and metastasis in patient disease and outline a potential therapeutic strategy for targeting these cells.

## Materials and methods

### Cell lines

The following breast cancer cell lines were purchased from ATCC (Manassas, VA, USA): HCC38, MDA-MB-436, MCF-7, T47D, BT474, MDA-MB-468, BT-20, HCC1187, Hs578T, BT549, and MDA-MB-231. LM2-4 cells, a highly metastatic variant of the MDA-MB-231 cell line was a gift from Robert Kerbel. All cell lines were tested and shown to be free of mycoplasma. The HCC38, BT549, and LM2-4 cells were further authenticated by short tandem repeat (STR) testing. Cells used in mice were additionally tested and found to be negative for an extensive panel of mouse pathogens. Cell lines were cultured in complete DMEM medium (DMEM supplemented with 10% fetal bovine serum (FBS) + 1% L-glutamine, sodium pyruvate, non-essential amino acids, and antibiotic/antimycotic).

### Cell transfection and lentiviral transduction

Plasmids containing enhanced specificity Cas9 and the appropriate guide RNA’s in the pLentiCRISPRv2 vector were purchased from GenScript (Piscataway, NJ, USA) for generating stable knockout with lentivirus and selected using puromycin. Transient transfections for all CRISPR/Cas9 vectors into 293T cells were performed with Lipofectamine 3000 (Invitrogen, Thermo Fisher Scientific, Waltham, MA, USA), while HiPerFect Transfection Reagent (Qiagen, Hilden, Germany) was used for siRNAs. All transfections were performed according to the manufacturers’ instructions. FlexiTube siRNAs (Qiagen) included AllStars negative control, *ZEB1* (SI04339587), *WNT5A* (SI04384184), *DACT1* (SI00359275), *TGFBI* (SI02780722), *ALCAM* (SI02780155), and *TSPAN5* (SI04151665).

### Generation of CRISPR knockout cells

Stable knockout of select genes was achieved by transducing BT549 or LM2-4 cells with lentivirus vectors expressing enhanced specificity Cas9 and the appropriate guide RNA’s targeting human *TGFBI* or *ZEB1* (GenScript) and pooling puromycin-resistant cells. A vector lacking the guide RNA was used as a negative control. Successful knockout of the respective targets was verified by Western blot for *ZEB1* and Real-time QPCR for *TGFBI*.

### Flow cytometry

Single-cell suspensions were prepared from cultured HCC38 or SUM149 cells, blocked in 0.5% BSA/PBS, and stained with the following antibodies prior to sorting: CD49f-PE 1:10 (555736, GoH3; BD Biosciences, San Jose, CA, USA); EpCAM–Alexa 647 1:20 (324212, 9C4; BioLegend, San Diego, CA, USA); and αvβ3–biotin 1:40 (MAB1976B, LM609; MilliporeSigma, Burlington, MA, USA) and Streptavidin-Brilliant Violet 421 1:80 (BioLegend). Propidium iodide solution (0.5 μg/ml) was used to detect dead cells. Viable cells were collected by sorting with a FACSDiva or FACSAria machine (BD Biosciences). In some cases, differentiation assays were performed by culturing sorted cells for exactly 10 passages (6–7 weeks after sorting) before re-analyzing by flow cytometry. See Supplementary Information for gating strategies (Supplementary Fig. 7a–c).

### Bulk RNA-Seq

After sorting HCC38 cells, RNA was purified from an equal number of cells per experiment for each cell type (approximately 50–70,000 cells) using a RNeasy Mini Kit (Qiagen). The samples were then submitted to the IGM Genomics Core at UCSD for validation of RNA quality and sequencing was performed on a HiSeq 4000 (Illumina, San Diego, CA, USA).

### Bioinformatics analysis

RNA-seq data were analyzed with a pipeline implemented in the BCBio-nextgen workflow manager https://zenodo.org/record/4686097#.YRLzj4hKiM8. Briefly, we aligned reads to GRch37 reference genome using STAR^33^ and quantified expression levels with Salmon 0.13.1^34^. We then annotated genes with BioMART^35^, keeping only protein coding genes with more than one read count for analysis. DEG was then identified using DESEQ2^36^. Gene Set Enrichment analysis was performed using R package LIGER on the Hallmark and Reactome gene sets available in MSigDB^37^. The 20 gene signature was derived by overlap analysis between the two differential gene expression analyses followed by manual curation for gene set membership. The public gene expression profile from breast cancer cell lines^13^ was obtained at NCBI GEO (GSE50470) and the corresponding intrinsic subtype information obtained from Prat et al.^12^. The CSC signature score was calculated according to Barbie et al.^38^ implemented in the gseapy python package (v0.9.8).

### Real-time qPCR

qPCR experiments on cultured cells were performed by collecting total RNA using the RNeasy Mini Kit (Qiagen) and reverse transcribing with the High-Capacity cDNA Reverse Transcription Kit (Applied Biosystems, Thermo Fisher Scientific). Relative mRNA levels from sorted cells were examined using the Cells-to-CT kit (Life Technologies) according to the manufacturer’s instructions. Lysates were prepared from 90,000 freshly sorted HCC38 cells. Real-time qPCR was performed using iTaq Universal SYBR Green Supermix (Bio-Rad, Hercules, CA, USA) and run on a LightCycler 480 qPCR System (Roche, Basel, Switzerland). See Supplementary Methods for a list of primers.

### Immunoblotting

Whole cell lysates were prepared from cell lines with RIPA lysis buffer (100 mM Tris pH 7.5, 150 mM sodium chloride, 0.1% deoxycholate, 0.1% SDS, 50 mM NaF, Protease inhibitor cocktail (Roche), 2 mM PMSF, 2 mM sodium orthovanadate) combined with scraping and the lysates cleared by centrifugation. Standard Western blotting procedures were performed. The following primary antibodies were used for immunoblotting at a dilution of 1:1000: ZEB1 (3396, Cell Signaling Technology, Danvers, MA, USA), Full-length PARP (9532, Cell Signaling Technology), Hsp90 (sc-13119, Santa Cruz, Dallas, TX, USA) and β-actin (MABT825, MilliporeSigma). Treatments with rhTGFBI (R&D Systems, Minneapolis, MN, USA) or vehicle control (PBS) were performed at the specified doses for 24 h prior to harvesting lysates. All blots or gels derive from the same experiment and were processed in parallel. See Supplementary Information for unedited blots (Supplementary Fig. 8a–c).

### TGFBI ELISA

Conditioned media from BT549 and LM2-4 cell lines was collected after 48 h in phenol-free complete DMEM culture medium. Secreted TGFBI levels were then quantified with the Human TGFBI (BIGH3) ELISA kit (Invitrogen, Thermo Fisher Scientific) according to the manufacturer’s instructions. Conditioned media was diluted 1:50 (BT549) or 1:200 (LM2-4) in order to fit on the standard curve.

### Immunofluorescence staining

Immunofluorescence staining was performed on cultured cells in a 4-well chamberslide (Nunc, Thermo Fisher Scientific) fixed briefly in 2% paraformaldehyde in PBS at room temperature and permeabilized. All samples were blocked with 1:80 normal goat serum in 0.1% BSA/PBS before incubation in phospho-γH2AX primary antibody diluted 1:100 (9718, Cell Signaling Technology) overnight at 4 °C, followed by incubation with DAPI and 1:500 Alexa Fluor 647-conjugated secondary antibody (A32733, Invitrogen, Thermo Fisher Scientific) for 2 h at RT. Alternatively, proliferation was measured by incubating live cells with Edu for 2 h at 37 °C prior to labeling the Edu with Alexa Fluor 647 according to manufacturer’s instructions (Invitrogen, Thermo Fisher Scientific). DAPI was used to visualize nuclei. Cells stained by either method were imaged with a Nikon A1R confocal microscope and images captured from four randomly selected fields with a 60× objective. Micronuclei were then manually counted for each field or the percent positive cells quantified using Image J software.

### Cell viability

XTT cell viability assays were performed by first seeding cells into a 96-well tissue culture plate at the following density per well: LM2-4 (4,000), BT549 (2,000), HCC38 (4,000), or MDA-MB-436 (7,500). After cells attached overnight the indicated concentrations of Olaparib (Lynparza) (Selleckchem, Houston, TX, USA) or vehicle alone (DMSO) were then added to the wells in 100 μL phenol-free complete DMEM medium. After 48 h, XTT substrate (Thermo Fisher Scientific) was added to the wells and incubated for 2 h before reading the A450 nm on a plate reader. Cell viability for each of the indicated treatments was expressed as a percent of the vehicle control wells.

### Copy number alterations

Genomic DNA was prepared from 5 × 10^5^ cells with the DNeasy Blood and Tissue Kit (Qiagen) and submitted to the IGM Genomics Core at UCSD. CNA was determined by hybridizing 200 ng of DNA per sample onto an Infinium CoreExome-24 Array (Illumina) and analyzing with Nexus CN software (version 7.5). The log2 ratio of signal intensity were calculated for each SNP on the array and copy number segments identified using the Bioconductor copynumber package^39^, including winsorization to remove outliers, imputation of missing value, and multi-sample copy number segmentation using *multipcf*. The differences in log2 ratio of all larger segments (more than 10 kb) were compared between control and KO BT549 cells.

### Tumorsphere assays

Primary tumorsphere formation was assessed in cells grown under anchorage-independent conditions in methylcellulose. BT549 (15,000) or LM2-4 cells (10,000) were cultured in 0.9 mL of 1% methylcellulose/complete DMEM medium in ultra-low adhesion 24-well dishes (Corning, Corning, NY, USA) and cells cultured for 10−12 days. Primary tumorspheres were assessed by counting colonies consisting of at least 6 cells from 4 fields per well with a 10× objective. We measured self-renewal by collecting primary tumorspheres by dilution in at least 3 volumes of PBS, dissociating them with trypsin for approximately 10 min, and re-seeding in 1% methylcellulose before evaluating secondary colonies after 10 days. For treatment with rhTGFBI (R&D Systems), a single dose of 500 ng/mL was added only once, when initially embedding the cells, and compared against cells receiving the same volume of vehicle (PBS).

### Orthotopic breast cancer

Tumors were generated by injection of a limiting number of sorted HCC38 cells diluted in 50 μl sterile PBS and injected into the inguinal fat pads of 8- to 10-week-old adult female nonobese diabetic/severe combined immunodeficiency/interleukin-2 receptor γ chain knockout (NSG) mice (purchased from UCSD Animal Care Program colony). All mice were monitored weekly for tumor formation by gentle palpation. Most tumors formed within 8−10 weeks. Tumor volume was measured with calipers twice weekly by a blinded observer. The experiment was concluded, and mice were sacrificed just prior to the tumors reaching the maximum allowable size of 2 cm^3^ or 14 weeks, whichever came first. Estimated tumor-initiating cell frequencies were calculated with the Extreme Limiting Dilution (ELDA) web-based tool^40^ http://bioinf.wehi.edu.au/software/elda/. Primary tumor mass was determined by assessing the wet weight of the resected tumors. The remaining tumor-free mice were harvested at 14 weeks and the absence of any detectable tumor was confirmed by whole-mount staining.

### Ethics

All mouse studies described were approved by the UCSD Institutional Animal Care and Use Committee (IACUC) and performed in accordance with the guidelines set forth in the NIH’s Guide for the Care and Use of Laboratory Animals (National Academies Press, 2011).

### Statistics

Data presentation and statistical tests are indicated in the figure legends. Two-tailed Student’s t-tests were used for comparing two means while ANOVA was performed for 3 or more data sets. Post hoc analysis was performed using an appropriate multiple comparisons test as indicated in the legends. For all analyses, *P* < 0.05 was considered statistically significant. Statistical analysis was performed using GraphPad Prism software (San Diego, CA, USA).

See Supplementary Material for additional methods.

### Reporting summary

Further information on research design is available in the Nature Research Reporting Summary linked to this article.

## Supplementary information

Supplementary Material

Reporting Summary

## Data Availability

RNA sequencing data that support the findings of this study have been deposited in the NCBI Sequence Read Archive (SRA) with the primary accession code PRJNA750073.
