# Supplementary material for: Stem-like breast cancer cells in the activated state resist genetic stress via TGFBI-ZEB1
Source: NPJ Breast Cancer. 2022 Jan 13;8:5. doi: 10.1038/s41523-021-00375-w (PMC8758745; doi:10.1038/s41523-021-00375-w)
Supplement: Supplementary file 1 — Supplementary Material [file 41523_2021_375_MOESM1_ESM.pdf]

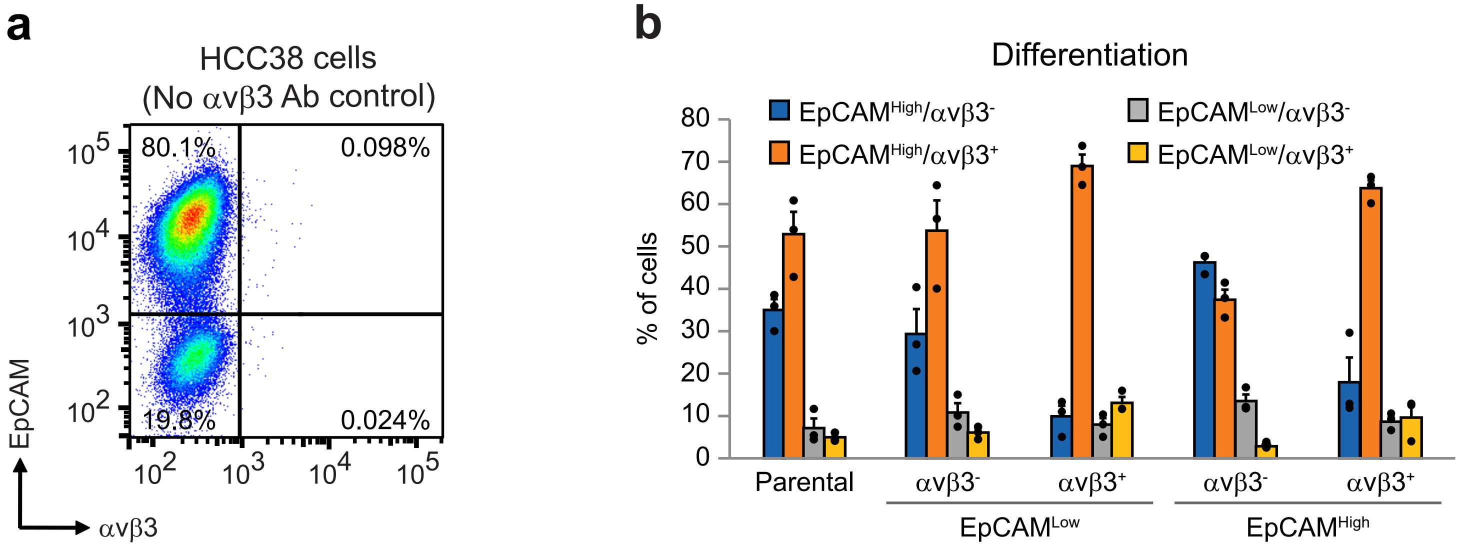

Supplementary Figure 1. αvβ3 expression is associated with a stem-like subset of HCC38 cells. Related to Figure 1. (**a**) Representative FACS density plot showing the minus αvβ3 antibody staining control for HCC38 cells from the same experiment as shown in Figure 1a. (**b**) Graphical summary of the data from Figure 1d showing differentiation of each sorted HCC38 cell type after 6 weeks (10 passages). n=3 independent experiments. Data represent the mean ± s.e.m.

a

Expression of mammary cell markers
($\alpha v\beta 3^+$ vs $\alpha v\beta 3^-$)

	Adjusted P-value	
**Basal/stem**	EpCAM^{Low}/$\alpha v\beta 3^+$	EpCAM^{High}/$\alpha v\beta 3^+$
TAGLN	0.00000065	1.27E-29
ACTA2	0.01278342	2.85E-17
SNAI2	0.00696259	0.00000026
CDK1	2.47E-17	5.74E-13
TP63	0.00044293	0.00004445
MYLK	0.00007346	0.00000001
CAV1	0.00001066	0.00149390
**Luminal progenitor**		
KIT	9.49E-22	3.30E-14
ELF5	0.00139219	0.14411442

b

Differentially Expressed Genes (DEG)
($\alpha v\beta 3^+$ vs $\alpha v\beta 3^-$)

		EpCAM^{High}		
		Down	No Change	Up
EpCAM^{Low}	Down	30	74	0
	No Change	113	13,481	223
	Up	3	177	245

c

Gene set enrichment (GSEA)
($\alpha v\beta 3^+$ vs $\alpha v\beta 3^-$)

	Adjusted P-value	
**Luteal gene sets**	EpCAM^{Low}/$\alpha v\beta 3^+$	EpCAM^{High}/$\alpha v\beta 3^+$
Hypoxia	0.00537607	0.00626033
Response to abiotic stimulus	0.00032372	0.00172383
EMT	0.00106292	0.00090348
Tissue development	0.00032372	0.00026557
Regulation of cell death	0.00184790	0.00026557
Response to oxygen levels	0.00076907	0.04564985
Response to external stimulus	0.01415284	0.00026557
Cellular response to stress	0.00032372	0.00026557
**Follicular gene sets**		
Myc targets V1	0.00106292	0.00090348
Posttranscriptional regulation of gene expression	0.00032372	0.00172383
Ribonucleoprotein complex biogenesis	0.00032372	0.00026557
mRNA processing	0.00106292	0.00090348
Ribosome biogenesis	0.00032372	0.00026557

Supplementary Figure 2. Association of EpCAM^{Low}/$\alpha v\beta 3^+$ status with stem-like cells in the activated state. Related to Figure 2. (**a**) Adjusted P-values for the data in Figure 2b showing the relative expression of select markers of mammary stem/basal and luminal progenitor cell types in $\alpha v\beta 3^+$ versus $\alpha v\beta 3^-$ cells. (**b**) Table depicting the number of differentially expressed genes in each cell type, as represented in Figure 2c. Selection criteria was ≥1.5-fold change in gene expression and $P<0.05$. (**c**) Adjusted P-values for the data in Figure 2d comparing $\alpha v\beta 3^+$ vs $\alpha v\beta 3^-$ cells in select gene sets enriched in stem/basal cells during the luteal (Active) or follicular (Inactive) menstrual cycle phases. (**a-c**) Statistics by Student's t-test with Benjamini-Hochburg multiple comparisons test. (**a-c**) n=3 independent experiments.

a

Gene Set Enrichment (GSEA)
(EpCAM^{Low}/αvβ3^{+} vs αvβ3^{-})

**Cell Function**	Adj. *P*-value
CGP_Schuetz Breast Cancer Ductal Invasive UP (*POSTN*, *TGFBI*, *DACT1*, *ZEB1*, *PLAUR*)	0.000095
CGP_Lim Mammary Stem Cell UP (*POSTN*, *VGLL3*, *PRICKLE1*, *AMOTL1*)	0.000095
CGP_Wong Adult Tissue Stem Module (*POSTN*, *DLC1*, *ALCAM*, *LTBP2*, *WNT5A*)	0.000705
**Signaling Pathways**	
CGP_Koinuma Targets of Smad2 or Smad3 (*TGFBI*, *DACT1*, *ALCAM*, *LTBP2*, *PTHLH*, *AMOTL1*)	0.000095
CGP_Kim WT1 Targets UP (*ZEB1*, *PLAUR*, *PTHLH*, *IL11*, *COBL*)	0.000095
CGP_Plasari TGFB1 Targets UP (*PLAUR*, *IL11*, *PTHLH*)	0.000173
GO_Negative Regulation of Canonical Wnt (*WNT5A*, *PRICKLE1*, *DACT1*, *SFRP4*)	0.022596

b

EpCAM^{Low}/αvβ3^{+} Gene Signature

**vs. all 3**	**Log2 Fold Change**	**Adj. P-value**
VGLL3	1.40	0.01072518
PRICKLE1	0.76	0.00303213
TSPAN5	1.22	0.00013199
COBL	1.12	0.04970365
POSTN	2.25	0.00923573
FREM2	0.67	0.00303213
RSAD2	1.17	0.01072518
RBPMS2	0.90	0.04640431
ZEB1	2.24	0.01618511
DACT1	2.36	0.00714717
TGFBI	0.72	0.04179088
DLC1	1.90	0.01625919
LTBP2	0.56	0.02288825
ALCAM	1.19	0.03571846
**vs. αvβ3^{-}**		
WNT5A	1.16	0.01664855
SFRP4	2.10	0.00886863
IL11	1.06	0.00130237
PLAUR	0.81	0.00637551
PTHLH	2.69	0.00005395
AMOTL1	0.63	0.00020870

Supplementary Figure 3. Unique genes associated with EpCAM^{Low}/αvβ3^{+} cells. Related to Figure 3. (**a**) Adjusted P-values for select gene sets enriched in EpCAM^{Low}/αvβ3^{+} cells. Specific genes (from DEG) responsible for the enrichment score are listed underneath the name of the gene set. (**b**) Log2 fold change and adjusted P-values for select genes unique to EpCAM^{Low}/αvβ3^{+} cells after the indicated comparisons. (**a,b**) Statistics by Student's t-test with Benjamini-Hochburg multiple comparisons test. n=3 independent experiments.

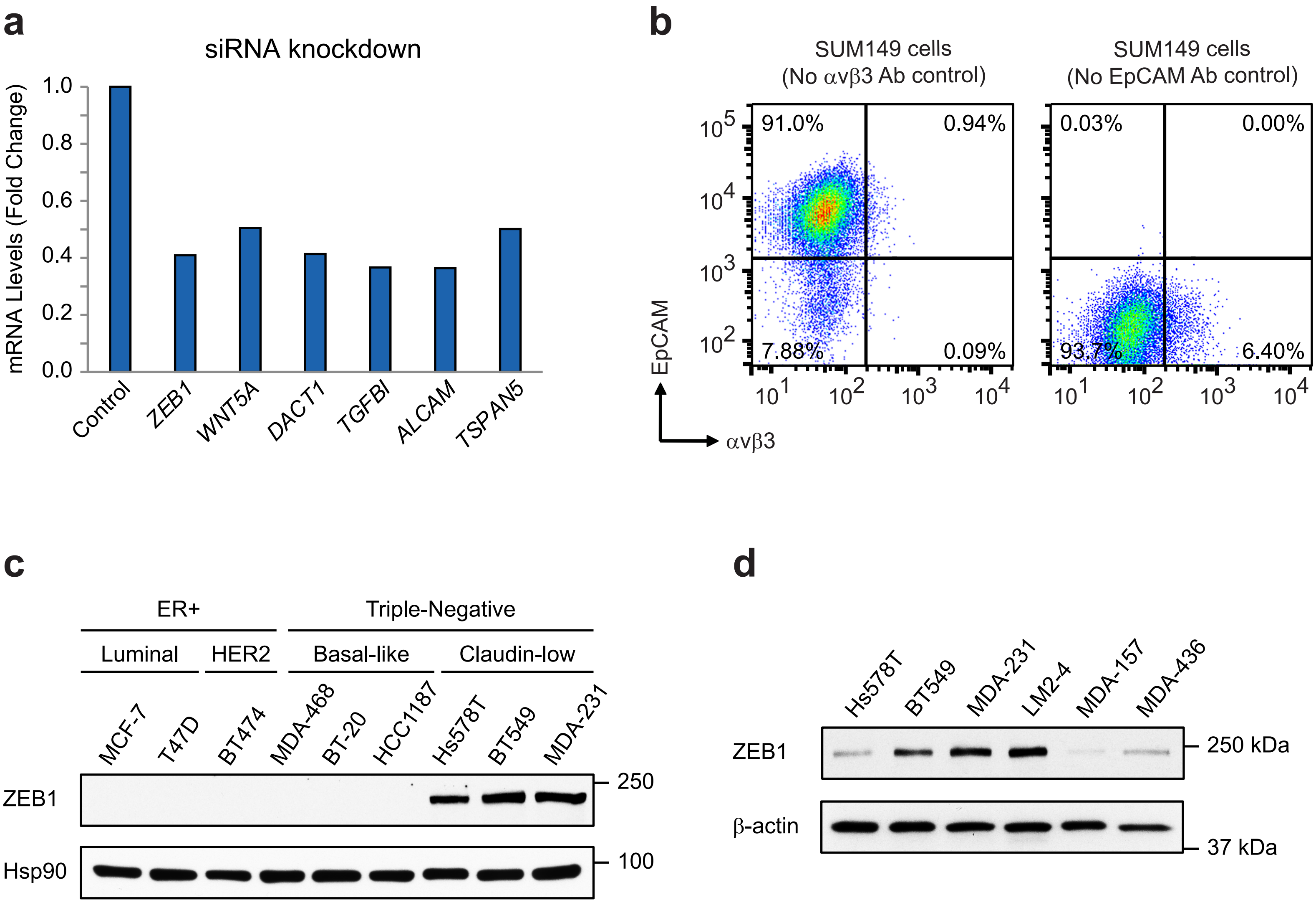

Supplementary Figure 4. Validation controls for siRNA screen and SUM149 FACS experiments. Related to Figure 4. (**a**) QPCR experiment showing efficient knockdown of the indicated target genes after transfection with siRNA. (**b**) FACS density plots of the minus αvβ3 and EpCAM antibody staining controls for SUM149 cells from the same experiment as shown in Figure 4d. (**c**) Representative immunoblot of lysates from a panel of breast cancer cell lines. (**d**) Immunoblot comparing ZEB1 expression in a panel of claudin-low breast cancer cell lines including LM2-4 cells, a metastasis-enriched variant of the MDA-MB-231 cell line. Hsp90 (**c**) or β-actin (**d**) are shown as loading controls. (**c,d**) Molecular weight markers are indicated in kilodaltons. (**a-d**) n=3 independent experiments.

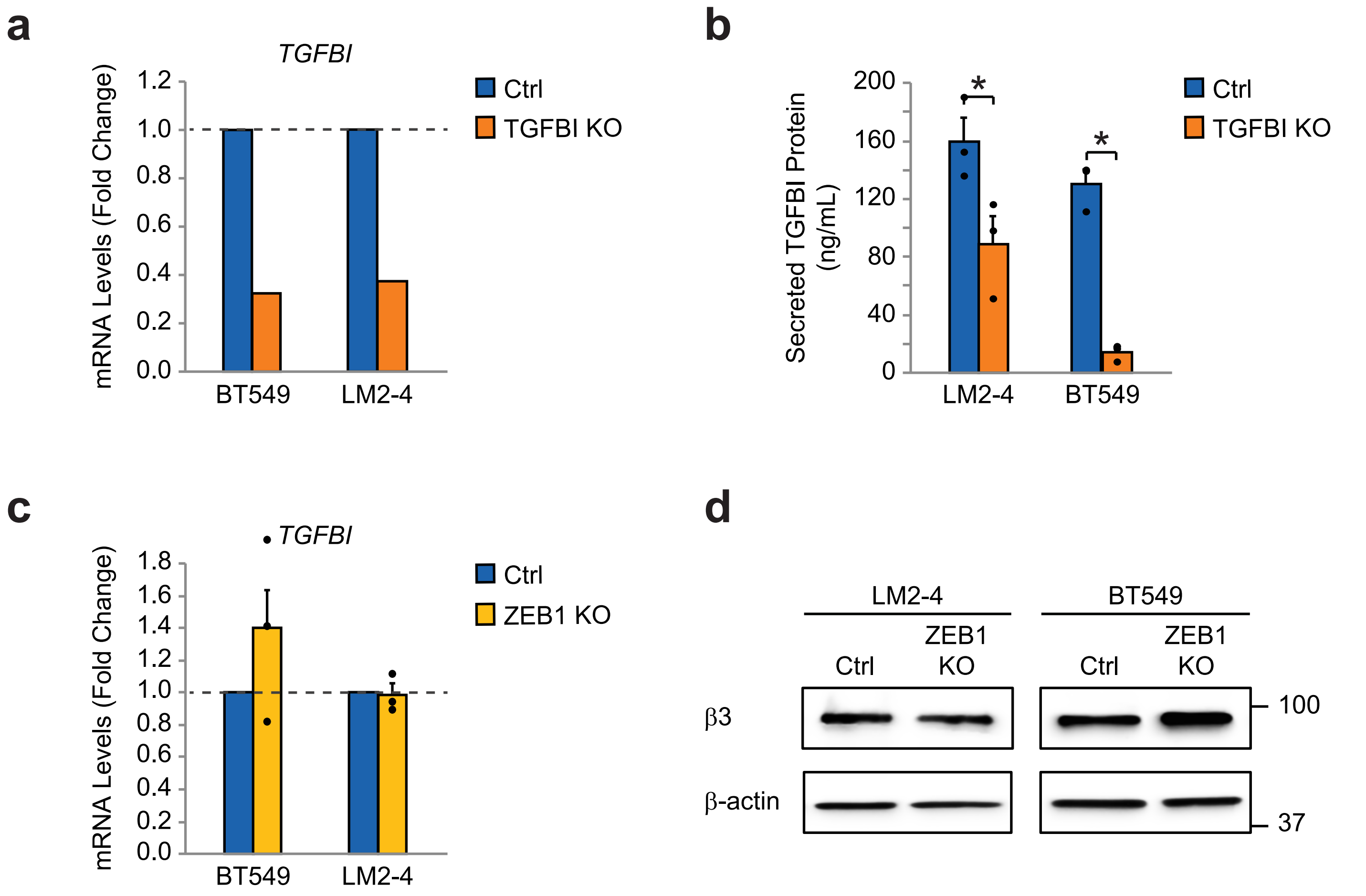

Supplementary Figure 5. Controls for CRISPR knockout cells. Related to Figure 5. (**a,b**) Validation of reduced mRNA (**a**) and secreted protein levels (**b**) after CRISPR/Cas9 gene excision of *TGFBI* in the indicated cell types (TGFBI KO) compared to control cells (Ctrl). (**a**) Representative QPCR experiment showing relative levels of *TGFBI* mRNA. (**b**) ELISA for secreted TGFBI protein levels in 48 hr conditioned media. Statistics by student's t-test. *P<0.05. (**c**) QPCR experiments showing no significant changes in levels of *TGFBI* mRNA due to deletion of ZEB1. (**a,c**) All samples were run in duplicate with GAPDH as a loading control. (**b,c**) Data represent the mean ± s.e.m. (**d**) Representative immunoblot for the β3 integrin subunit in control and ZEB1 knockout cell lines. β-actin is shown as a loading control. Molecular weight markers are indicated in kilodaltons. (**a-d**) n=3 independent experiments.

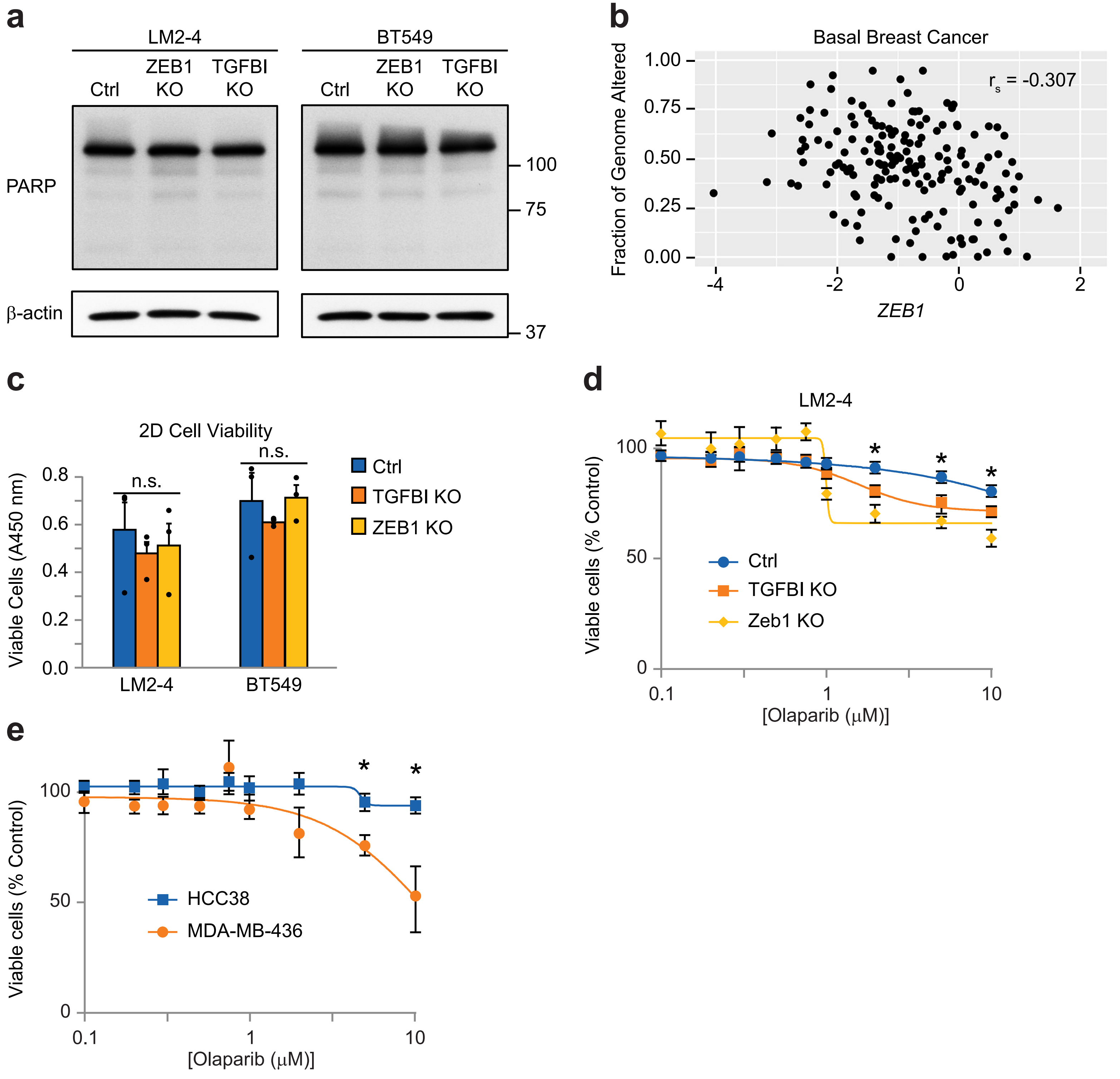

Supplementary Figure 6. Deletion of TGFBI or ZEB1 enhances chromosomal instability and synergizes with Olaparib treatment. Related to Figure 6. (**a**) Representative immunoblots showing no differences in the relative amounts of cleaved PARP (89 kDa) compared to full-length PARP (116 kDa). Molecular weight markers are indicated in kilodaltons. (**b**) TCGA analysis of basal breast cancers for ZEB1 gene expression and the fraction of the genome altered. Statistics performed by Spearman rank correlation with bonferroni multiple comparisons test. P=0.00054. n=171 different cancers, each represented by a black dot. (**c-e**) XTT cell viability assays. (**c,d**) The effect of TGFBI or ZEB1 deletion on cell viability in 2-dimensional (2D) adherent culture conditions (**c**) or sensitivity to the PARP inhibitor Olaparib (**d**) in the indicated cell types. (**e**) Comparison of Olaparib sensitivity in resistant (HCC38) and sensitive (MDA-MB-436) cell types at the same doses used in (**d**). (**d,e**) Curves are plotted relative to vehicle controls for each group and fitted by non-linear regression. (**a,c-e**) n=3 independent experiments. (**c-e**) Data represent the mean ± s.e.m. Statistics by one-way (**c**) or two-way (**d,e**) ANOVA with Dunnett's (**c,d**) or Sidak's (**e**) multiple comparisons test. *P<0.05 for 2, 5, and 10 μM Olaparib (TGFBI or ZEB1 KO versus control) (**d**) and 5 and 10 μM Olaparib (MDA-MB-436 versus HCC38) (**e**). n.s. = not significant.

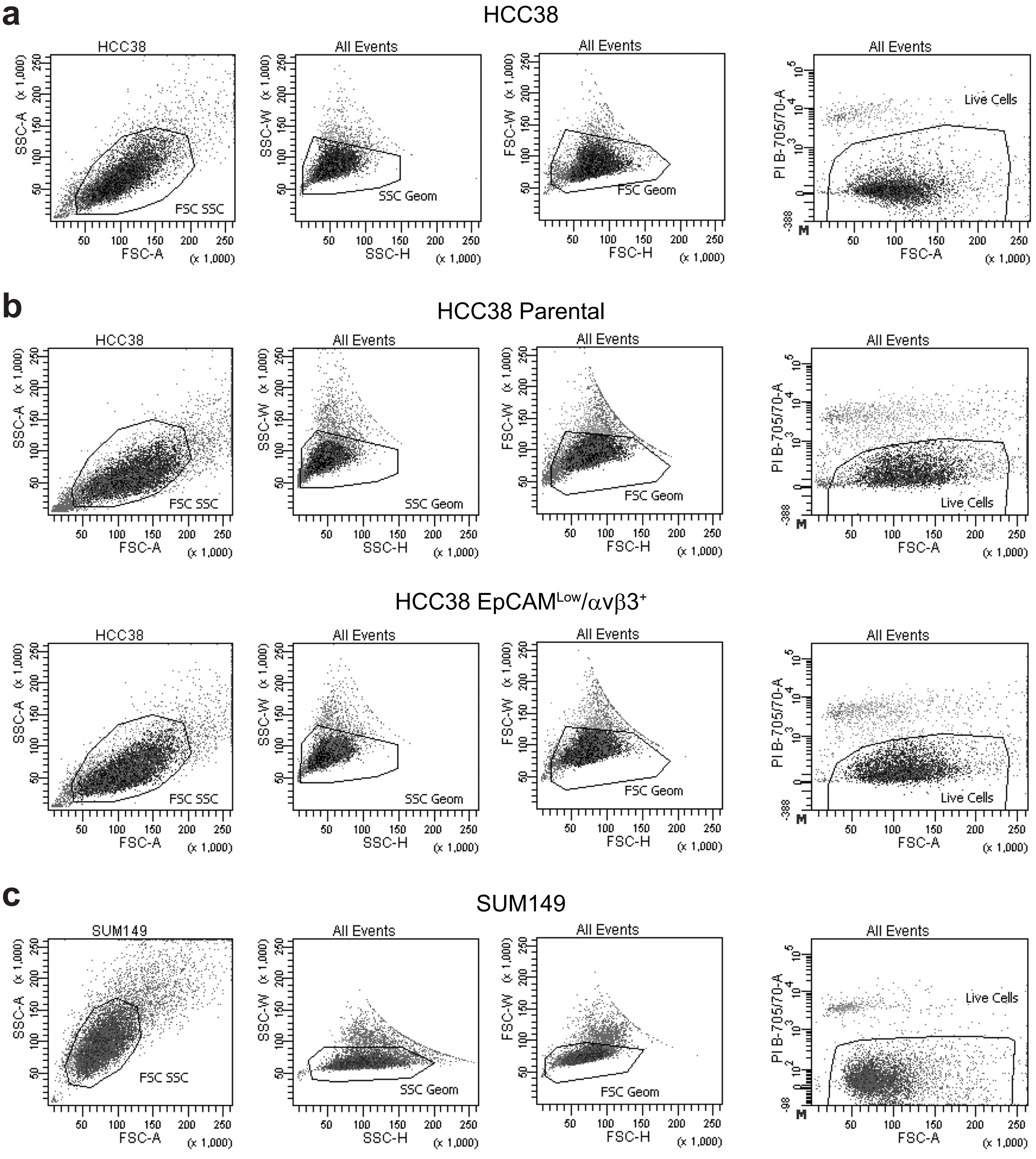

Supplementary Figure 7. FACS gating strategies. (**a-c**) Sequential gating strategies for the representative FACS plots shown in figures. (**a**) HCC38 cells in figure 1a. (**b**) Parental HCC38 cells (**top**) and sorted EpCAM^{Low}/αvβ3^{+} cells (**bottom**) in figure 1d. (**c**) SUM149 cells in figure 4d.

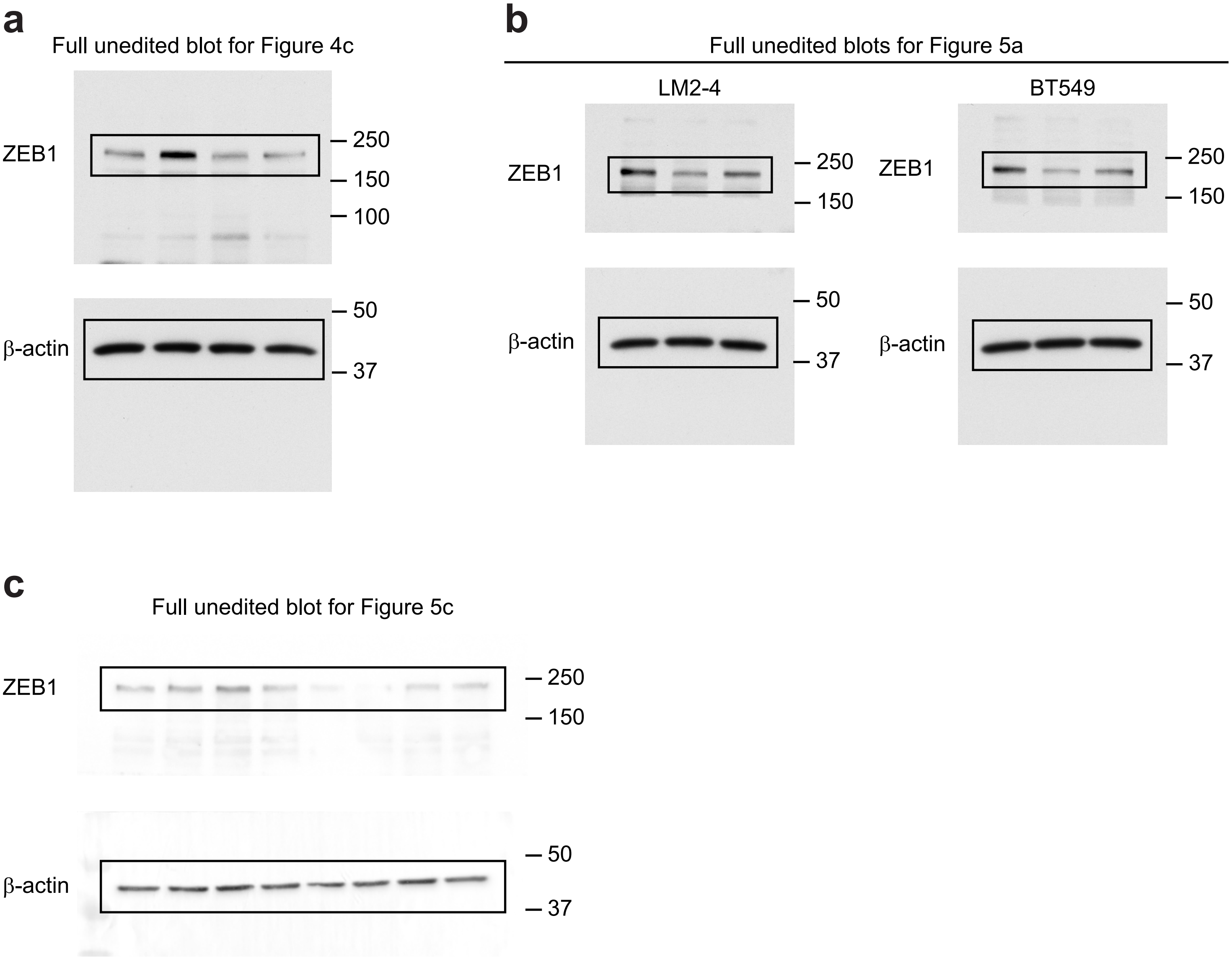

Supplementary Figure 8. Unedited Western blots. Unprocessed scans for the immunoblot data shown in main figures 4c (**a**), 5a (**b**) and 5c (**c**). Boxes outline the areas cropped for use in figures. Molecular weight markers are indicated in kilodaltons.

Supplementary Methods

Real-time qPCR

qPCR Primers

Primer Name	Primer Sequence (5'-3')
**Human**	
hZEB1-F	GATGATGAATGCGAGTCAGATGC
hZEB1-R	ACAGCAGTGTCTTGTTGTTGT
hWNT5A-F	ATTCTTGGTGGTCGCTAGGTA
hWNT5A-R	CGCCTTCTCCGATGTACTGC
hDACT1-F	TTGAACTGTTTGAGGCGAAGAG
hDACT1-R	ACTGAACACCGAGTTAGAGGAAT
hTGFBI-F	CACTCTCAAACCTTTACGAGACC
hTGFBI-R	CGTTGCTAGGGGCGAAGATG
hALCAM-F	TCCTGCCGTCTGCTCTTCT
hALCAM-R	TTCTGAGGTACGTCAAGTCGG
hTSPAN5-F	GGGTCCTGAAGTCAGTTGTTG
hTSPAN5-R	ATGGAAGAGATGTTGGACAGAAC
