# Supplementary material for: Stem-like breast cancer cells in the activated state resist genetic stress via TGFBI-ZEB1
Source: NPJ Breast Cancer. 2022 Jan 13;8:5. doi: 10.1038/s41523-021-00375-w (PMC8758745; doi:10.1038/s41523-021-00375-w)
Supplement: Supplementary file 2 — Reporting Summary [file 41523_2021_375_MOESM2_ESM.pdf]

nature portfolio

Corresponding author(s): Jay Desgrosellier

Last updated by author(s): Nov 18, 2021

Reporting Summary

Nature Portfolio wishes to improve the reproducibility of the work that we publish. This form provides structure for consistency and transparency in reporting. For further information on Nature Portfolio policies, see our Editorial Policies and the Editorial Policy Checklist.

Statistics

For all statistical analyses, confirm that the following items are present in the figure legend, table legend, main text, or Methods section.

n/a	Confirmed	
☐	☒	The exact sample size (*n*) for each experimental group/condition, given as a discrete number and unit of measurement
☐	☒	A statement on whether measurements were taken from distinct samples or whether the same sample was measured repeatedly
☐	☒	The statistical test(s) used AND whether they are one- or two-sided<br>*Only common tests should be described solely by name; describe more complex techniques in the Methods section.*
☐	☒	A description of all covariates tested
☐	☒	A description of any assumptions or corrections, such as tests of normality and adjustment for multiple comparisons
☐	☒	A full description of the statistical parameters including central tendency (e.g. means) or other basic estimates (e.g. regression coefficient) AND variation (e.g. standard deviation) or associated estimates of uncertainty (e.g. confidence intervals)
☐	☒	For null hypothesis testing, the test statistic (e.g. *F*, *t*, *r*) with confidence intervals, effect sizes, degrees of freedom and *P* value noted<br>*Give P values as exact values whenever suitable.*
☒	☐	For Bayesian analysis, information on the choice of priors and Markov chain Monte Carlo settings
☒	☐	For hierarchical and complex designs, identification of the appropriate level for tests and full reporting of outcomes
☒	☐	Estimates of effect sizes (e.g. Cohen's *d*, Pearson's *r*), indicating how they were calculated

Our web collection on statistics for biologists contains articles on many of the points above.

Software and code

Policy information about availability of computer code

Data collection	Microsoft Excel
Data analysis	Microsoft Excel and GraphPad Prism

For manuscripts utilizing custom algorithms or software that are central to the research but not yet described in published literature, software must be made available to editors and reviewers. We strongly encourage code deposition in a community repository (e.g. GitHub). See the Nature Portfolio guidelines for submitting code & software for further information.

Data

Policy information about availability of data

All manuscripts must include a data availability statement. This statement should provide the following information, where applicable:

- Accession codes, unique identifiers, or web links for publicly available datasets
- A description of any restrictions on data availability
- For clinical datasets or third party data, please ensure that the statement adheres to our policy

RNA sequencing data that support the findings of this study have been deposited in the NCBI Sequence Read Archive (SRA) with the primary accession code PRJNA750073 (https://www.ncbi.nlm.nih.gov/sra/PRJNA750073).

Field-specific reporting

Please select the one below that is the best fit for your research. If you are not sure, read the appropriate sections before making your selection.

☒ Life sciences ☐ Behavioural & social sciences ☐ Ecological, evolutionary & environmental sciences

For a reference copy of the document with all sections, see nature.com/documents/nr-reporting-summary-flat.pdf

Life sciences study design

All studies must disclose on these points even when the disclosure is negative.

Sample size	For in vitro assays we used a sample size of 3-4 independent experiments to evaluate statistical differences. For in vivo experiments, each condition was tested in at least 2 independent experiments with 2-3 animals per condition. These sample sizes were chosen since they have previously been shown to be sufficient to detect statistical differences in prior experiments.
Data exclusions	No data was excluded.
Replication	All findings were verified in multiple independent experiments (See sample size). All replication attempts were successful.
Randomization	For in vivo studies, mice were randomized by body weight prior to injecting tumor cells. For in vitro studies with stable cell lines, experiments were performed with pooled populations to prevent clonal bias.
Blinding	For experiments in mice, blinding was readily achieved by having one individual perform the injections, referring to each mouse only by code, and another different individual responsible for detecting and measuring the tumors. We used a similar technique for blinding our tumorsphere assays in vitro, where one individual would set-up the experiment, coding each well, and one or two different individuals would count the colonies.

Reporting for specific materials, systems and methods

We require information from authors about some types of materials, experimental systems and methods used in many studies. Here, indicate whether each material, system or method listed is relevant to your study. If you are not sure if a list item applies to your research, read the appropriate section before selecting a response.

Materials & experimental systems

n/a	Involved in the study	
☐	☒	Antibodies
☐	☒	Eukaryotic cell lines
☒	☐	Palaeontology and archaeology
☐	☒	Animals and other organisms
☒	☐	Human research participants
☒	☐	Clinical data
☒	☐	Dual use research of concern

Methods

n/a	Involved in the study	
☒	☐	ChIP-seq
☐	☒	Flow cytometry
☒	☐	MRI-based neuroimaging

Antibodies

Antibodies used	ZEB1 (clone D80D3; cat# 3396; Cell Signaling Technology, Danvers, MA, USA), Full-length PARP (clone 46D11; cat# 9532; Cell Signaling Technology), Hsp90 (clone F8; cat# sc-13119; Santa Cruz, Dallas, TX, USA), β-actin (clone 4C2; cat# MABT825; MilliporeSigma, Burlington, MA, USA), phospho-gammaH2AX (clone 20E3; cat# 9718; Cell Signaling Technology), CD49f-PE (clone GoH3; cat# 555736; BD Biosciences, San Jose, CA, USA), EpCAM–Alexa 647 (clone 9C4; cat# 324212; BioLegend, San Diego, CA, USA) and αvβ3–biotin (clone LM609; cat# MAB1976B; MilliporeSigma)
Validation	In addition to the antibody validation shown on the manufacturer's website, we provide appropriate positive and negative controls to verify the specificity of our antibody staining in the manuscript. To confirm our antibody staining results, we also measured mRNA levels by QPCR as an additional validation step.

Eukaryotic cell lines

Policy information about cell lines

Cell line source(s)	HCC38, MCF-7, T47D, BT474, MDA-MB-468, BT-20, HCC1187, Hs578T, BT549, and MDA-MB-231 breast cancer cell lines were purchased from ATCC (Manassas, VA, USA). LM2-4 cells, a highly metastatic variant of the MDA-MB-231 cell line was a gift from Robert Kerbel.

Authentication	STR testing was performed on HCC38, BT549 and LM2-4 cells.
Mycoplasma contamination	All cell lines were tested and shown to be free of mycoplasma. Cells used in mice were additionally tested and found to be negative for an extensive panel of mouse pathogens.
Commonly misidentified lines (See ICLAC register)	No commonly misidentified cell lines were used in this study.

Animals and other organisms

Policy information about studies involving animals; ARRIVE guidelines recommended for reporting animal research

Laboratory animals	This study used laboratory mice (mus musculus). Specifically we used 8- to 10-week-old adult female nonobese diabetic/ severe combined immunodeficiency/ interleukin-2 receptor gamma chain knockout (NSG) mice (purchased from UCSD Animal Care Program colony).
Wild animals	This study did not involve wild animals.
Field-collected samples	This study did not involve samples collected from the field.
Ethics oversight	All mouse studies described were approved by the UCSD Institutional Animal Care and Use Committee (IACUC) and performed in accordance with the guidelines set forth in the NIH's Guide for the Care and Use of Laboratory Animals (National Academies Press, 2011).

Note that full information on the approval of the study protocol must also be provided in the manuscript.

Flow Cytometry

Plots

Confirm that:

☒ The axis labels state the marker and fluorochrome used (e.g. CD4-FITC).

☒ The axis scales are clearly visible. Include numbers along axes only for bottom left plot of group (a 'group' is an analysis of identical markers).

☒ All plots are contour plots with outliers or pseudocolor plots.

☒ A numerical value for number of cells or percentage (with statistics) is provided.

Methodology

Sample preparation	Single-cell suspensions were prepared from cultured HCC38 or SUM149 cells, blocked in 0.5% BSA/PBS, and stained with the following antibodies prior to sorting: CD49f-PE (GoH3; BD Biosciences, San Jose, CA, USA); EpCAM–Alexa 647 (9C4; BioLegend, San Diego, CA, USA); and αvβ3–biotin (LM609; MilliporeSigma, Burlington, MA, USA) and Streptavidin-Brilliant Violet 421 (BioLegend). Propidium iodide solution (0.5 µg/ml) was used to detect dead cells.
Instrument	Viable cells were collected by sorting with a FACSDiva or FACSAria machine (BD Biosciences).
Software	Data from flow cytometry experiments was analyzed with FlowJo software.
Cell population abundance	The abundance of the relevant cell populations is provided in the manuscript. The purity of our post-sort populations is best indicated by the consistent gene expression differences noted within these fractions from multiple experiments by RNA-Seq, including validation of the markers used for sorting (See principal component analysis in Figure 2a).
Gating strategy	Appropriate single-stain and fluorescence minus one (FOM) controls were used to draw all gates. Examples of our gating strategy are provided in the supplement to support the FACS plots shown in the main figures.

☒ Tick this box to confirm that a figure exemplifying the gating strategy is provided in the Supplementary Information.
